# Intimate partner violence against women. Does violence decrease after the entry of the alleged offender into the criminal justice system?

**DOI:** 10.1080/20961790.2021.1960616

**Published:** 2021-08-28

**Authors:** Paulo Vieira-Pinto, José Ignacio Muñoz-Barús, Tiago Taveira-Gomes, Maria João Vidal-Alves, Teresa Magalhães

**Affiliations:** a Department of Forensic Sciences, Pathology, Gynaecology and Obstetrics, Paediatrics, Institute of Forensic Sciences, University of Santiago de Compostela, Santiago de Compostela, Spain; b IINFACTS - Institute of Research and Advanced Training in Health Sciences, Department of Sciences, CESPU, University Institute of Health Sciences (IUCS), Gandra, Portugal; c Faculty of Medicine, University of Porto, Porto, Portugal; d CINTESIS - Centre of Health Technology and Service Research, University of Porto, Porto, Portugal; e Fernando Pessoa University, Porto, Portugal

**Keywords:** Forensic sciences, intimate partner violence, women, re-entry, recidivism, criminal justice system

## Abstract

Intimate partner violence (IPV) is simultaneously assumed as a serious crime and a major public health issue, having recurrences as one of its main characteristics and, consequently, re-entries of some alleged offenders in the criminal justice system (CJS). The main goal of this study is to assess if in cases of female victims of IPV, violence decreases after the first entry of the alleged offender in the CJS. A retrospective study was performed based on the analysis of police reports of alleged cases of IPV during a 4-year period. The final sample (*n* = 1 488) was divided into two groups according to the number of entries in the CJS (single or multiple) followed by a comparative approach. Results suggest that violence decreases after the first entry of alleged offenders in the CJS. Re-entries were found in only 15.5% of the cases but they were accountable for 3.3 times more crimes on average. Besides, victims of recidivism presented more injuries and required more medical care. Thus, a small group of alleged offenders seems to be more violent and accountable for most of the IPV crimes registered in the CJS suggesting that regardless of legal sanctions aiming to deter violence, these measures may not be enough for a certain group of offenders. This study sustains the need for a predictive model to quantify the risk of repeated IPV cases within the Portuguese population.

## Introduction

### Intimate partner violence

Intimate partner violence (IPV) is a troublesome and challenging occurrence worldwide and is simultaneously considered a public health issue and a social hazard with financial implications [1, 2]. Data from the World Health Organization (WHO) show that 30% to 38% of all women who have been in an intimate relationship have experienced physical and/or sexual violence from their intimate partners [3]. In the European Union, 22% of female citizens have reported physical and/or sexual violence, 43% have reported psychological violence and 55% have reported sexual harassment [4].

In Portugal, IPV is protected under a broader criminal concept of domestic violence (DV) and since 2001 has had a public nature, which means that the public prosecutor’s office may initiate criminal proceedings, regardless of whether the victim wishes to file a complaint, and the crime can be reported by anyone.

IPV is associated with two relevant aspects that must be considered: (1) the revictimization process and (2) violence escalation. Albeit the importance of these aspects, no prior research was found concerning how these aspects are addressed by the Portuguese criminal justice system (CJS).

Revictimization relates to the reoccurrence/repetition of violence inflicted by the same offender to the same victim. The actual amount of repetition of violence may remain unknown to the CJS if not reported and is difficult to measure since re-abuse episodes are often kept in secrecy by female victims due to, for example, shame, emotional attachment to the abuser or the belief that the abuser will change. Since the actual number of repetitions may remain unknown to the CJS if IPV events are underreported, it can be measured by analysing the re-entries of the offender into the CJS, which are the IPV events known by authorities. An evaluation of the risk of reoccurrence that includes the prior IPV cases seems to be fundamental to decrease and to prevent both fatal and nonfatal IPV-related cases [5]. However, professionals may fail in this risk assessment task and also in the detection of IPV indicators [6], as victims tend to conceal violence episodes, mainly from health professionals [6, 7] and police [8, 9].

In addition to its prevalence, some authors state that IPV displays an increasing tendency, both in frequency and severity [3, 10]. Interestingly, Bland and Ariel [8], in 36 000 police records of DV, between 2009 and 2014, found no escalation in most cases (76% had no second calls). Yet, their study reveals that the small group that recidivates is responsible for more severe violence, registering more harm caused [11, 12].

### The present paper

The main goals of this study are to provide know­ledge to prevent and combat IPV against women reoccurrence, test if the previously accepted theory that IPV is recurrent and worsens over time, as well as assess the impact of the CJS on these cases. Notwithstanding the fact that violence may exist in intimate relationships regardless of the sex of the involved persons [13, 14], in this paper, we focus on male-to-female IPV because studies show that these are the most frequently reported [1, 15].

The specific goals are to (1) identify indicators of future recidivism in the first offence, (2) identify violence severity (through harm measure), (3) identify differences between dyads with repeated incidents and a single report, and (4) determine whether IPV decreases after the alleged offender’s first entry into the CJS.

### CJS intervention

The CJS intervention is crucial in order to prioritize and follow-up cases according to the occurrence’s characteristics and the identifiable risk factors. It becomes clear that collecting as much knowledge as possible of each case is the only way to succeed in providing protection and reparation to IPV victims and their families.

Besides, there is some evidence suggesting that victims may not always report their intimate partner’s violence and may experience multiple offences before contacting authorities [16]. This endangers the safety of the victims and hampers a thorough assessment by the police, and adds a challenge.

The literature [17] systematically identifies a history of IPV as the main risk factor for intimate partner homicide (IPH), and several studies [5, 18–20] have shown that in a considerable percentage of IPH cases, previous episodes of physical violence have occurred. Therefore, there seems to be a consensus that early and correct identification of IPV cases may help prevent future revictimization.

To fully understand IPV events and their severity and escalation, it is necessary to study the following variables: (1) the type of violence suffered by the victims; (2) the number of injuries and their seve­rity, including the need for medical treatment; (3) the frequency of the offences; and (4) the number of entries of the alleged offenders into the CJS [8].

Based on the referred literature, this study intends to test the following hypothesis: (1) that IPV occurrences tend to decrease after the first entry of the alleged offender into the CJS due to the deterrence effect of the contact with the CJS (e.g. being notified, arrested, and publicly exposed) and (2) that differences are expected concerning the severity of harm displayed by single report victims and victims of repeated violence. Ultimately, it is expected that the results of this study provide knowledge to the CJS decision-makers and help prioritize CJS intervention in IPV cases against women according to their characteristics.

## Methods

A retrospective analysis was conducted regarding DV crimes, specifically the IPV perpetrated by men against women.

The IPV cases included in the present study were selected regardless of human differences such as socioeconomic status, race, ethnicity, language, nationality, sex, gender identity, sexual orientation, religion, geography or ability and occurred in nor­thern Portugal in Porto District, which presents one of the highest IPV rates in Portugal [21]. This study was reviewed and approved by the Health Ethics Committee of the Centro Hospitalar de S. João/Faculdade de Medicina da Universidade do Porto (S. João Hospital Center/Faculty of Medicine of the University of Porto).

Data were collected from the DV database of one of the Portuguese security forces, the Guarda Nacional Republicana (GNR). The inclusion criteria were (1) cases of alleged IPV (between intimate partners whether former or current and whether in dating, marital, or other similar relationships with or without cohabitation) [22]; (2) complaint(s) presented by the victim, always regarding the same offender, to GNR police stations in Porto; (3) a female victim aged 16 or older; (4) an alleged male offender aged 16 or older since persons under the age of 16 cannot be held criminally liable; (5) full identification of the victim and the alleged offender to allow tracking re-entry cases in the CJS within the same region; (6) an offender who had no prior criminal IPV-related records filed for at least 365 days before the current complaint; and (7) at least 365 days of follow-up.

**Figure 1.**
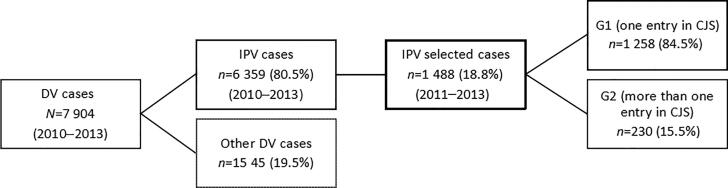
Cases selection on the domestic violence (DV) database of the Guarda Nacional Republicana (from 1 January 2010 to 31 December 2013). IPV: intimate partner violence; CJS: criminal justice system. G1: group 1; G2: group 2.

The DV database was scanned for cases occurring within a 4-year period (from 1 January 2010 to 31 December 2013). Thus, the case follow-up duration varied between 1 and 3 years to guarantee a broad range of analysis. The scan yielded 7 904 alleged DV cases, of which 6 359 (80.5%) corresponded to alleged IPV. In total, 1 488 (18.8%) cases met the full inclusion criteria, as described in Figure 1.

**Figure 2.**
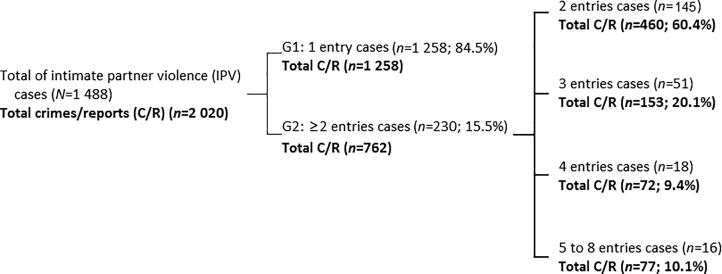
Number of entries of the alleged offender in the Criminal Justice System (CJS) and crimes reported in the analysed period. (from 1 January 2010 to 31 December 2013). G1: group 1; G2: group 2

For the present study, the sample was divided into two groups according to the number of entries into the CJS during the follow-up period: group 1 (G1) includes cases with one entry (*n* = 1 258) and group 2 (G2) includes cases with two or more entries (*n* = 230) (Figure 2). A comparative study of both groups was then performed considering the characteristics of the victims, alleged offenders, and violence. To compare G1 and G2, we considered the report of the first entry into the CJS. For injury analysis, we used the official Portuguese classification, which divides the cases into three categories: (1) absence of injuries, (2) minor injuries, and (3) severe injuries.

Statistical analysis was conducted using the R programming language [23]. In addition to the descriptive analysis, hypothesis testing for the association of characteristics with re-entry was carried out using the Chi-square test. The pattern of missing data was assessed using Little’s missing completely at random (MCAR) test using the LittleMCAR function [24]. The analysis was performed on imputed data using the multivariate imputation by chained equations (MICE) package [25]. Categorical variables were imputed using a proportional odds model, and continuous variables were imputed using unconditional mean imputation. All variables presented were considered in the imputation model. Data imputation was repeated 100 times. To assess the robustness of the results, the same analysis was performed with the removal of incomplete cases relevant to each test. Significance was considered at *P* < 0.05.

## Results

### Population characterization

The average age for victims was 40.9±11.5 years old (mean±SD, 17–82) in G1 and 39.9±11.2 years old (18–82) in G2, respectively. The alleged offenders were 42.9±11.8 years old (18–82) in G1 and 42.4±11.6 years old (19–90) in G2, respectively. There were no signifi­cant differences on the studied variables in victims,such as age, relationship with alleged offenders, employment status and economical dependency, between the two groups (Table 1), nor were there significant differences on variables in alleged offenders, including age, employment status, and risk factors such as alcohol/drug abuse and weapons possession, between the two groups (Tables 2). Other variables regarding risk factors were not analysed because GNR records did not contain such data.

**Table 1.** Victims’ characterization (*N* = 1 488)

Victims’ characterization	*n* (%)		P*
	G1 (*n* = 1 258)	G2 (*n* = 230)	
Age (years)			
≥16–20	22 (1.8)	5 (2.2)	0.477 (0.463)
21–30	215 (17.1)	41 (17.8)	
31–40	404 (32.1)	86 (37.4)	
41–50	378 (30.1)	55 (23.9)	
51–60	149 (11.8)	27 (11.7)	
61–64	27 (2.2)	6 (2.6)	
≥ 65	44 (3.5)	5 (2.2)	
Missing	19 (1.5)	5 (2.2)	–
Relationship with the alleged offender			
Past	217 (17.2)	31 (13.5)	0.189 (0.189)
Current	1 041 (82.8)	199 (86.5)	
Married	786 (62.5)	141 (61.3)	n.a.
In-union	250 (19.9)	57 (24.8)	
Dating	5 (0.4)	1 (0.4)	
Employment status			
Unemployed	475 (37.8)	79 (34.4)	0.392 (0.422)
Employed	463 (36.8)	97 (42.2)	
Retired	80 (6.4)	13 (5.7)	
Others	58 (4.6)	8 (3.5)	
Missing	182 (14.5)	33 (14.4)	–
Economical dependency			
Yes	339 (27.0)	61 (26.5)	0.821 (0.934)
No	834 (66.3)	146 (63.5)	
Missing	85 (6.8)	23 (10.0)	–

* P values presented for both imputed (former) and raw models (latter) with there were missing data.

**Table 2.** Alleged offenders’ characterization (*N* = 1 488)

Alleged offenders’ characterization	*n* (%)		P*
	G1 (*n* = 1 258)	G2 (*n* = 230)	
Age (years)			
≥16–20	7 (0.6)	2 (0.9)	0.620 (0.578)
21–30	145 (11.5)	24 (10.4)	
31–40	330 (26.2)	72 (31.3)	
41–50	342 (27.2)	59 (25.7)	
51–60	181 (14.4)	28 (12.2)	
61–64	23 (1.8)	7 (3.0)	
≥65	57 (4.5)	9 (3.9)	
Missing	17 (13.8)	29 (12.6)	–
Employment status			
Unemployed	485 (38.6)	90 (39.1)	0.714 (0.704)
Employed	551 (43.8)	95 (41.3)	
Missing	222 (17.7)	45 (19.6)	–
Risk factors			
Alcohol abuse			
Yes	646 (51.4)	121 (52.6)	0.860 (0.972)
No	564 (44.8)	104 (45.2)	
Missing	48 (3.8)	5 (2.2)	–
Drug abuse			
Yes	72 (5.7)	10 (4.4)	0.401 (0.461)
No	113 (90.5)	215 (93.5)	
Missing	48 (3.8)	5 (2.2)	–
Weapons possession			
Yes	145 (11.5)	26 (11.3)	0.637 (0.825)
No	868 (69.0)	168 (73.0)	
Missing	245 (19.5)	36 (15.7)	–

* P values presented for both imputed (former) and raw models (latter) because missing data exist.

### Entries into the CJS

In the studied sample, 15.5% (*n* = 230) of the alleged offenders re-entered the CJS (G2). Of this total, 22.2% (*n* = 51) had a third re-entry, 7.8% (*n* = 18) registered a fourth re-entry, and 7.0% (*n* = 16) had more than four re-entries with a maximum of eight. Thus, between 2011 and 2013, these individuals alone were responsible for 762 re-entries, thus reoffending on average 3.3 times more than G1 offen­ders (Figure 2).

### Violence characterization

In both groups, psychological and physical abuse were the most frequent forms of violence, and when isolated, psychological abuse was the most frequent in G1 (Table 3). Psychological violence was more frequent in G1 than in G2, and physical violence was more frequent in G2 than in G1, but with no significant differences (*P*=0.107 and *P*=0.486, respectively). No correlation was found between injuries and age or between injuries and the status of the victim-alleged abuser relationship as current or past.

**Table 3.** Violence at the first intimate partner violence (IPV) incident

Violence at IPV incident	*n* (%)		P*
	G1 (*n* = 1 258)	G2 (*n* = 230)	
Types of violence registered			
Physical	848 (67.4)	161 (70.0)	0.486
Psychological/emotional	906 (72.0)	153 (66.5)	0.107
Economical	98 (7.8)	19 (8.3)	0.912
Social isolation	108 (8.6)	14 (6.1)	0.255
Sexual	20 (1.6)	2 (0.9)	n.a.
Injuries			
No injuries	608 (48.3)	85 (37.0)	0.005 (0.017)^†^
Minor injuries	573 (45.6)	127 (55.2)	
Severe injuries	7 (0.6)	0 (0.0)	
Missing	70 (5.6)	18 (7.8)	–
Need for medical treatment			
Yes	157 (12.5)	42 (18.3)	0.024 (0.024)
No	1 101 (87.5)	188 (81.7)	
Need for hospitalization			
Yes	2 (0.2)	1 (0.4)	n.a.
No	1 210 (96.2)	217 (94.4)	
Missing	46 (3.6)	12 (5.2)	–

*P values presented for both imputed (former) and raw models (latter) when there were missing data; ^†^test for no injuries *versus* minor and severe injuries

## Discussion

### Recurrence and re-entries into the CJS

The present analysis does not allow us to know all the recurrence situations that may have happened in the selected cases but only the occurrences that have been reported to GNR in the period and region of the study (recidivism). In fact, the study design, according to the available data, only assures that at least in the previous year and in Porto District, any report and entry into the CJS has occurred. Consequently, recurrence may have occurred in G1 cases as well as in G2, where many more offences than reports may have happened. This, among other factors, is a result of the underreporting of cases due to the following: (1) ambi­valent feelings that lead the victims to conceal the abuse (e.g. feelings of shame and guilt; a belief that some forms of violence are acceptable; a fear of retaliation, of having economic difficulties or of losing child custody; a lack of support; a belief that the offender will change; negative experiences with the CJS that have caused a loss of belief in its effectiveness) [26–29]; (2) the absence of proper abuse detection by professionals [6]; and (3) persisting abuse tolerance by family and acquaintances [30].

Thus, the discussion of this study must take into consideration that the analysis exclusively considered the number of re-entries into the CJS and not the actual rate of recurrence, which is unknown (a dark figure of DV crime). Addressing re-entries allows us to better understand the impact of the CJS intervention as a deterrent of recidivism.

In the present study, only 15.5% of the total alleged offenders (*n* = 1 488) had re-entered the CJS, suggesting that the recurrence rate may be much higher. There is evidence that prosecution has a more discouraging effect on violent behaviours than the conviction itself [31]. Barnham et al. [11] support this by pointing out a significant decrease in IPV repetition when the alleged offender has contact with the CJS, refraining from abusing the victim for the following 2 years, as stated by other authors [8, 32]; our results are also similar (84.5% did not re-enter at least in the 1-year follow-up).

The decrease in re-entries over time may be due to the CJS intervention and/or to the few reports from victims (Figure 2). However, it is important to point out that prosecutions are frequently dismissed given the difficulty or even impracticality of proving the existence of a crime [2, 33, 34], many times due to the absence of the victim’s collaboration. Other studies consider that re-offences decrease over time even without legal intervention or rehabilitation [11, 35].

### Violence evolution

Despite the mere 15.5% of re-entries, it is notable that this rate corresponds to 230 alleged offenders (G2) who were responsible for a total of 762 cases of IPV crimes during the studied period (3.3 times more on average than G1, Figure 2). These alleged offen-ders continued to abuse despite the previous reports made by the victims (3 times or more in 37% of the cases: *n* = 762). Such a result is in accordance with the evidence provided by Bland and Ariel [8], who found that most of the harm is caused by a small fraction of alleged offenders. This suggests that for this group of individuals, the general measures applied by the CJS may not be enough to deter violence, while the majority of individuals seem to respond adequately to the CJS intervention. In fact, according to several authors, regardless of legal reforms, sanctions designed to deter repeat offenders are unable to hamper future aggressions [15, 29, 34, 36].

Regarding the types of violence, no differences were found for aggressions between the groups (Table 3), probably because physical violence is always the most frequent type present in cases that are reported to the police (it is easier to prove in a court of law).

However, considering physical harm, the victims in first entries in G2 had more injuries than those in G1 (*P* = 0.005) and more often needed medical assistance (*P* = 0.024). This supports the theory that violence severity is higher in recidivism cases and tends to increase in intensity and severity over time [8, 11]. This increases the danger for victims, dimini­shes self-efficacy perception and control loci in victims, and increases the difficulty of leaving abusive relationships or reporting [27, 36].

The current data do not allow a deeper analysis of violence severity. The injury evaluation made by the police is based on an official tool, the *Standardized Domestic Violence Report* (SDVR), created by a specialized workgroup following the *II National Plan Against Domestic Violence* (Council of Ministers Resolution 88/2003). It focuses exclusively on visible physical injuries at the time of the report (the most affected visible anatomical areas are the face, head, neck, upper torso and hands) [6, 37]; hidden injuries are included if reported by the victims. This method does not suffice for injury research studies due to (1) the lack of knowledge of police officers to describe injuries (a clinical task); (2) the lack of perceivability of many injuries; and (3) the limitation to two levels of injury (minor or severe). However, in on-the-scene reporting, this is the only tool that police officers have. In addition, victims may be taken to a hospital and/or forensic medical service, where injuries can be described in more detail, namely, using scales such as the *Injury Severity Score* (ISS) or its abbreviated version (AIS) [38].

The justification for the use of the police eva­luation tool in this study, rather than a medical tool, is consistency since not all victims are seen by a physician. In addition, the goal was to estimate the number of injured women rather than injury severity itself. Physical violence was found to be mostly minor, as found in other studies [6, 39], while emotional and verbal abuse, despite its lasting consequences on victims’ health [6, 40], was not systematically measured.

### Case profiling

Even though we assessed all available data regarding IPV cases, no statistical associations were found between re-entry and victims’ and alleged offenders’ backgrounds. This may suggest that either the variables collected are individually of little relevance to assess the likelihood of recidivism or that a multivariate relationship is present. Known risk factors, such as adverse childhood experiences, previous experience of DV, DV perpetration or other criminal background and history of physical and mental illness [20], were not collected. These aspects might allow predictive models to quantify the risk of recidivism in each case [41].

### Study limitations and avenues for further reviews

The limitations/weaknesses that can be identified in the current study are mainly related to the use of available data. There are relevant variables that were not available for analysis, primarily concerning risk factors and injury descriptions. In addition to the absence of information in some variables reducing the strength of our estimations, the frequencies in G2 for some categories were low and limited the statistical analysis.

Data were collected from the database of one of the two Portuguese security forces and exclusively concern Porto District. However, the data depict 17 out of the district’s 18 municipalities, and the district is the second largest and has one of the highest IPV rates in Portugal.

The follow-up time of cases varied between 1 and 3 years. This may slightly bias the characterization of recidivism since repeat offences may have occurred immediately before or after the studied period and may not have been considered.

The lack of a thorough injury classification should be addressed in the future to improve the analysis of harm severity and allow a reabuse risk assessment.

Future studies should consider the development of solutions to manage existing resources in the CJS to improve the current one-size-fits-all solution. It would be important to understand the effectiveness of the existing mechanisms in the CJS, namely, as far as the Portuguese CJS is concerned, by evaluating (1) the ability of the Provisional Suspension of Criminal Proceedings to reduce recidivism of IPV and (2) the satisfaction and trust of the women victims in the effectiveness of the CJS.

Finally, a predictive model for quantifying the risk of repeated IPV cases within the Portuguese population is needed to provide help before more severe or even fatal occurrences take place. Such a model would be extremely helpful not only for professionals but also for those who endure such unte­nable realities.

## Conclusion

The present study indicates that violence may decrease after the first entry of the alleged offender in the CJS. In fact, in 84.5% of the studied cases, no re-entries were observed in the at least 1-year follow-up in the Porto District, according to the GNR database. This does not rule out unreported reabuses, reports to other police agencies, reports made in other regions of the country or the possibility of the same offenders having moved on to new victims.

A low rate of the alleged offenders had re-entered the CJS (15.5%). However, these individuals alone were responsible for 762 re-entries, reoffending on average 3.3 times more than those with only one entry.

This study found that reabused victims presented more injuries than single-abuse victims did (*P* = 0.005) and required more medical assistance (*P* = 0.024), suggesting that the violence was more severe in repeated violence cases.

This study further provided evidence that women presenting more severe physical harm at the first reported event are those who suffer higher rates of reabuse. This may allow the identification in the first report of dyads with a higher probability of IPV reoccurrence and those who may benefit from a more effective response to prevent revictimization. In fact, regardless of legal sanctions aiming to deter IPV, this may not be enough for some offenders.

No personal features or behavioural patterns related to re-entry cases were found with reabuse predictor potential.
